# Focus on Extracellular Vesicles: Development of Extracellular Vesicle-Based Therapeutic Systems

**DOI:** 10.3390/ijms17020172

**Published:** 2016-02-06

**Authors:** Shin-ichiro Ohno, Gregor P. C. Drummen, Masahiko Kuroda

**Affiliations:** 1 Department of Molecular Pathology, Tokyo Medical University, 6-1-1 Shinjuku, Shinjuku-ku, Tokyo 160-8402, Japan; s-ohno@tokyo-med.ac.jp; 2 Cellular Stress and Ageing Program, Bionanoscience and Bio-Imaging Program, Bio & Nano-Solutions, D-33647 Bielefeld, Germany

**Keywords:** exosome, microvesicle, extracellular vesicle, exosome, dexosome, outer membrane vesicle, stem cell, drug delivery system, vaccine, clinical trial

## Abstract

Many types of cells release phospholipid membrane vesicles thought to play key roles in cell-cell communication, antigen presentation, and the spread of infectious agents. Extracellular vesicles (EVs) carry various proteins, messenger RNAs (mRNAs), and microRNAs (miRNAs), like a “message in a bottle” to cells in remote locations. The encapsulated molecules are protected from multiple types of degradative enzymes in body fluids, making EVs ideal for delivering drugs. This review presents an overview of the potential roles of EVs as natural drugs and novel drug-delivery systems.

## 1. Introduction

Membrane vesicles secreted from various types of cells are categorized as exosomes, microvesicles (MVs), and apoptotic vesicles according to their size, pathway of origin, and constituent molecules [1,2]. Exosomes, which are 30–100 nm sized vesicles derived from the late endosome, can be isolated from the conditioned medium of various cells or from body fluids using a sucrose gradient (1.13–1.19 g/mL) or ultracentrifugation (100,000× *g* for 70 min) [3]. Exosomes are enriched in heat shock proteins (HSP70, HSP90), tetraspanin family molecules (CD9, CD63, CD81), and components of the ESCRT (endosomal sorting complex required for transport) machinery (e.g., Alix and TSG101) [1,2]. MVs, also called shedding vesicles, ectosomes or microparticles, are between 100 and 1000 nm in diameter and are enriched in phosphatidylserine, integrins, selectins, and CD40 ligand. Unlike exosomes, MVs are formed through outward budding of the plasma membrane [4,5]. Apoptotic vesicles are derived from apoptotic cells and are distinctly different from exosomes because they abundantly contain histones associated with membranes that float at high sucrose densities (1.24–1.28 g/mL) and because they are very heterogeneous in size and morphology when observed by EM [6].

**Figure 1:**
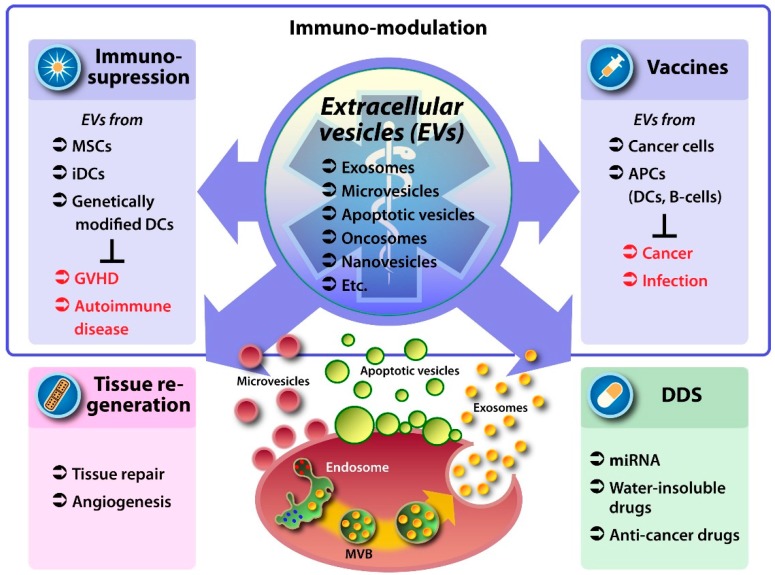
Research aimed at developing extracellular vesicles (EVs) for clinical applications. APC: Antigen presenting cell; DCs: Dendritic cells; iDCs: Immature dendritic cells; DDS: Drug delivery system; GVHD: Graft-versus-host disease; MSCs: Mesenchymal stem cells.

Because the methods used to isolate and purify membrane vesicles differ significantly between studies, we do not strictly distinguish these categories in this review, but instead collectively refer to such vesicular structures as “extracellular vesicles” (EVs) and explicitly identify the subtype when necessary. EV proteins that are expressed on lipid bilayer membranes stimulate receptors on the surfaces of physically separated cells, and the encapsulated materials play functional roles in the cells that take up the EVs. These characteristics raise the possibility that EVs might be used therapeutically. Research aimed at applying EVs in a clinical setting can be divided into two broad categories (Figure 1): (1) EVs as biological medicines, *i.e.*, therapeutics that take advantage of the positive effect exerted by molecules contained in the EVs secreted by particular types of cells.

The majority of these efforts are focused on immune-regulatory effects (*i.e.*, immune suppression or immune activation) in the context of vaccines (Figure 1, upper). In addition, EVs have been reported to mediate effects on tissue repair and angiogenesis (Figure 1, lower left); (2) EVs as carriers for drug-delivery systems (DDSs), taking advantage of EVs’ natural characteristics to deliver molecules to target cells (Figure 1, lower right). In this review, we summarize the development of EV-based drugs and drug-delivery systems, and discuss future possibilities for clinical applications of EVs. A comprehensive introduction to EVs is provided in this focus edition in the article by Kalra *et al.* [2].

## 2. EVs as Therapeutic Vehicles

### 2.1. Liposomes vs. EVs

**Figure 2:**
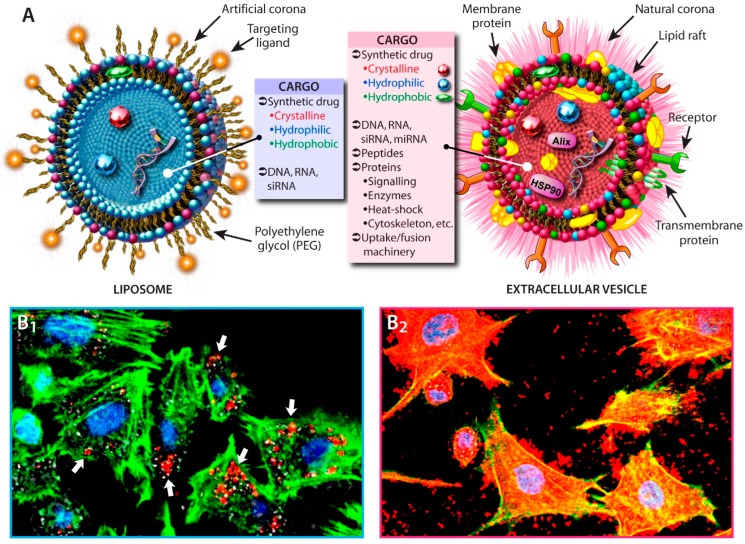
Comparison of liposomes and extracellular vesicles (EVs) as drug-delivery-systems (DDS). (**A**) Schematic depiction of lipid-based vesicular carriers (not to scale). Note that in comparison to liposomes, EV membranes not only consist of a diverse number of lipid classes, but also contain trans-membrane and membrane-associated proteins, receptors, adhesion molecules, as well as a natural corona. Such features must be engineered into liposomes to obtain a stable and suitable DDS; (**B**) Accumulation of exosomes is profoundly higher (B_2_) than accumulation of artificial nano-carriers such as liposomes (B_1_; white arrows) in PC12 neuronal cells after 24 h incubation [12]. Red (Dil, C_59_H_97_ClN_2_O_4_): exosomes or nano-carriers; Green (rabbit anti-PGP9.5): actin microfilaments; Blue (DAPI): nuclei.

Historically, artificial vesicular carriers, e.g., liposomes (Figure 2A), have been used to carry therapeutics to target tissues and cells. These liposomes generally have various sizes with either single or multiple lipid bilayers, e.g., Small or Large Unilamellar (SUVs; LUVs) *vs*. Multilamellar Vesicles. The lipid compositions of liposomes generally vary, depending on their intended application, but most commonly phosphatidylcholine (lecithin) and phosphatidylethanolamine or derivatives are used in combination with cholesterol to control membrane fluidity and prevent physical instability and premature content leakage (high permeability). Depending on the polarity of the therapeutic agent, either encapsulation in the liposomal lumen or incorporation into the lipid bilayer occurs. At least 19 drugs with liposomal DDSs have been approved by the FDA and EMA [7,8], whilst nearly 450 phase I‒III open interventional studies are currently being conducted [9]. Despite several advantages, liposomes suffer from drawbacks that include problems related to stability, fatty acyl moiety oxidation, drug loading, liposome targeting, drug leakage and release, and others. Most importantly, intravenous injection of liposomal drugs has been known to induce complement activation-related pseudoallergy (CARPA) in some cases; an acute and severe hypersensitivity reaction [10,11].

Unlike liposomes, EVs have the distinct advantage that their membranes are structurally comparable to other membranous structures found in cells. This means that both their lipid composition, fluidity, the presence of membrane proteins, potential targeting and docking modalities are all preserved in EVs (Figure 2A). Furthermore, EVs are likely to carry the required fusogenic properties or uptake machinery within their corpora. In comparison, liposomes are primitive vesicular structures, over which some measure of control regarding their properties can be obtained through control of their size, lipid composition, corona, and addition of potential targeting peptides or other surface modifications. However, the difficulty of obtaining suitable coronas that ensure sufficiently decreased clearance by the mononuclear phagocyte system (long circulation times), prevent opsonization (fouling with complement, immunoglobulins, fibronectin and apolipoproteins) and non-specific interactions with cells illustrates the relative primitiveness of liposomes as DDSs compared with EVs. For instance, a common way to reduce surface fouling is by coating with polyethylene glycol (PEG). However, corona design requires significant engineering, as recent research into nano-sized DDS shows that for long circulation times and anti-fouling both mixed length PEG chains with particular conformations and a sufficiently high PEG chain density are required [13,14]. Although PEGylation might reduce the aforementioned limitations, it also potentially reduces interaction of the DDS with target cells, negatively affecting the DDS’s biodistribution. Furthermore, Armstrong and co-worker report that preexisting PEG antibodies are present in 22%–25% of healthy blood donors as a consequence of exposure to PEG in cosmetics and foodstuffs [15,16], which might negatively influence the effectiveness of therapeutic interventions [17]. Nonetheless, PEGylation is the only coating strategy for DDSs that is regarded as safe for use in humans and carries the GRAS classification (Generally Recognized As Safe) issued by the FDA [18]. Overall and because of the nature and structural composition of EVs, these should be considered superior as DDSs compared with liposomes and consequently open up new ways for drug delivery.

### 2.2. Cellular Uptake and Drug-Loading

Cellular uptake of EVs surpasses that of more traditional carriers such as liposomes or nanoparticles (compare Figure 2B1,2). Batrakova’s group not only demonstrated unparalleled accumulation of drug-loaded exosomes compared with 100 nm poly(lactic-co-glycolic acid) or liposomes in PC12 cells, but also that *ex vivo* catalase-loaded macrophage-derived exosomes significantly accumulated in mouse brain neurons and microglial cells upon intranasal administration [12], circumventing the limitations of various DDSs regarding mucosal and blood brain barrier traversal.

Fuhrman and collaborators recently compared various passive and active drug-loading methods, *i.e.*, electroporation, saponin treatment, extrusion and dialysis, and used porphyrins of various hydrophobicities as model drugs [19]. They determined that porphyrin-loaded EVs from MDA-MB231 breast cancer, human umbilical vein endothelial and human mesenchymal stem cells all showed superior cellular uptake and light-induced cellular killing than drug-loaded phosphatidyl-choline/cholesterol liposomes or free porphyrins. Furthermore, passive loading of hydrophobic compounds into EVs was significantly higher than in liposomes, which might be a result of the presence of particular domains within EV membranes that are absent in the artificial membranes of liposomes. Although it has long been known that, through the right combination of phospholipids and cholesterol in liposomal formulations, the properties of liposomal membranes can be steered, such artificial membranes are unlikely to be able to compete with the more natural structural properties of EV membranes (Figure 2A). Finally, Fuhrman *et al*. determined that of all nondestructive active loading methods, particularly the saponin-assisted method allowed high loading efficacies. Similar results were obtained in the aforementioned catalase-loaded exosomes; albeit that saponin treatment, sonication and extrusion all showed high loading efficiencies and no significant degradation of catalase [12]. Despite the reported high loading capacities, there are limits to the maximal attainable loading, in particular in exosomes, due to size restrictions and the native presence of numerous proteins and nucleic acids. For small RNAs, electroporation is generally used to load EVs, as established by Alvarez-Erviti and co-workers [20]. We also successfully used electroporation to load miRNA into exosomes targeted to the epidermal growth factor receptor (EGFR) of breast cancer cells [21]. However, electroporation has been known to induce aggregation in particular when loading siRNA and as a result the true siRNA content is often overestimated [22]. Beside the aforementioned loading methods, genetic manipulation of the parental cell has been used to incorporate particular proteins into EVs. This strategy can be realized by, for instance, introducing genetic material, e.g., cDNA, encoding the protein of interest and a protein that localizes in EVs such as C1C2 to the parental cell [23]. Alternatively, plasmid-based introduction has been successfully used to modify macrophages for reproduction, packaging, and targeted therapeutics delivery to treat neurodegenerative disorders [24]. Finally, Maguire *et al.* took it one step further and proposed exploiting viral packaging systems (from non-enveloped viruses) to improve gene delivery by creating hybrid vesicles called “vexosomes” (vector-exosomes) [25]. Although they achieved significantly better transfection and reduced immunogenicity compared with the free viral vector, such hybrid systems would require additional scrutiny.

Overall, the abovementioned research results show that drug-loaded EVs are superiors as DDS both in terms of loading, circulation times, and cellular uptake and payload delivery. Furthermore, due to the nature of EVs, therapeutic options built into EVs are significantly better protected from the harsh outside environment than other lipid-based DDS.

## 3. Medicinal Use of Native EVs

### 3.1. EVs from Mesenchymal Stem Cells

Mesenchymal stem cells (MSCs) are self-renewing precursor cells (multipotent stem cells) that are able to differentiate into a variety of cell types, including bone, cartilage, muscle, marrow, ligament, adipose and connective tissues [26,27]. Consequently, MSCs have predominantly been investigated in the context of clinical applications aimed at repairing damaged tissue. These studies revealed that MSCs exert tissue-repair functions in blood vessels, lung, kidney, bone, and cartilage tissue in various animal disease models [28]. In addition, MSCs have potent immunosuppressive activities that can inhibit both innate and adaptive immune responses. Research and clinical trials have been conducted to investigate the inhibitory effects of MSC transplantation on graft-versus-host disease (GVHD) after allogeneic tissue transplantation, rheumatism, uveitis, diabetes, and inflammatory bowel disease [29]. Furthermore, via their neuroprotective effects, MSCs can prevent amyotrophic lateral sclerosis, multiple sclerosis, Parkinson’s disease, and glaucoma [30,31,32,33,34]. As noted above, MSCs have multiple functions that include tissue repair, immunosuppression, and neuroprotection, but the mechanisms underlying these functions remain largely unknown. Recent work showed that administration of the exosome fraction isolated from MSCs has an effect similar to that of MSC transplantation [35]. Treatment of GVHD with MSC-derived EVs decreased the pro-inflammatory cytokine response of peripheral blood mononuclear cells (PBMCs) and diarrhea volume. In lung disease, EVs derived from bone marrow MSCs inhibit pulmonary arterial hypertension in rats [36] and acute lung injury induced by *E. coli* endotoxin in mice [37,38]. Tissue-repair functions are not limited to MSCs, but have also been reported in embryonic stem cell (ESC). Although transplantation of ESCs is considered very effective in vascular disorders, serious side effects such as teratomas may occur [39,40]. Nonetheless, Khan *et al*. successfully treated myocardial infarction (MI) in a MI mouse model without side-effects when administrating ESC-derived EVs [41]. EVs derived from MSCs also have useful functions beyond activation or suppression of immune responses. For example, EVs derived from MSCs protect against acute kidney injury and may activate a proliferative program in surviving tubular cells after injury via horizontal transfer of mRNA [42,43]. These results indicate that the identities and functions of MSCs are reflected in their EVs. Consequently, the EVs of MSCs have attracted attention as a novel cell-free approach for stem cell therapy of various diseases [44].

### 3.2. EVs from Antigen Presenting and Other Immune Cells

Antigen-presenting cells (APCs) include B-cells, macrophages, and dendritic cells (DCs) that display foreign antigens on their surfaces in association with major histocompatibility complex (MHC) proteins. These antigen-MHC complexes activate T-cells via T-cell receptors (TCRs). Recent work showed that EVs secreted from APCs also express MHCs and costimulatory receptors on their surfaces [45,46,47]. Moreover, these EVs can stimulate T-cells and activate the adaptive immune system. For example, EVs from peripheral blood of BALB/c mice infected with the reticulocyte-prone non-lethal *Plasmodium yoelii* strain 17X contain parasite proteins. Moreover, immunization of mice with purified EVs elicits IgG antibodies capable of recognizing *P. yoelii*-infected red blood cells and protects against lethal challenge with the normocyte-prone lethal *P. yoelii* strain 17XL [48]. Furthermore, peptide- or protein-loaded EVs derived from DCs (dexosomes) can inhibit tumor development in a CD4^+^ T- and B-cell-dependent manner [49,50]. Damo and co-workers recently showed that dexosomes produced with the Toll-like receptor 3 (TLR-3) agonist polyinosinic-polycytidylic acid (poly(I:C)) in conjunction with ovalbumin (OVA) stimulate OVA-specific CD8^+^ and CD4^+^ T-cell proliferation and acquisition of effector functions [51]. This effect was significantly higher compared with using lipopolysaccharide (LPS) for TLR-4 or CpG-B for TLR-9. Furthermore, when DCs were loaded with a B16F10 melanoma cell lysate to produce dexosomes containing tumor antigens, the resulting vaccine strongly induced activation of melanoma-specific CD8^+^ T-cells and recruitment of cytotoxic CD8^+^ T-cells, NK and NK-T-cells to subcutaneously grafted B16-F10 melanoma tumors in C57BL/6 mice. As a consequence, tumor growth was significantly more reduced and survival prolonged compared with comparable vaccines that lack the aforementioned pretreatments.

In the presence of IL-4 and IL-10, cultured bone marrow DCs develop into immunosuppressive DCs. Surprisingly, EVs derived from immunosuppressive DCs inhibit delayed-type hypersensitivity (DTH) and rheumatism in animal models [52]. This mechanism is still not well understood, but probably depends on MHC Class II and the CD95-CD95L signaling pathway. In addition to the immunosuppressive EVs generated by cultured cells *in vitro*, as described above, immunosuppressive EVs also exist *in vivo*. EVs purified from the serum or bronchoalveolar lavage fluid (BALF) of antigen-sensitized mice have immunosuppressive activity [53]. EVs from genetically modified bone marrow-derived DCs expressing IL-4 or FasL suppress inflammation of DTH in a mouse model of collagen-induced arthritis [54,55]. In addition, the proportion of Foxp3^+^CD4^+^CD25^+^ regulatory T-cells is elevated and production of IL-2 and IFN-γ is inhibited in human PBMCs incubated with EVs from breast milk [56].

Beside induction of immune responses by bioactive membrane constituents, microRNA (miRNA) luminal cargos have been assumed to mediate biological functions via gene regulation of the target cell. Although EV transfer of small RNAs have now been observed in various systems involving immune cells, the exact role of EVs in this transfer remains unclear. For instance, endogenous *miR-142* and *miR-223* from human macrophages were shown to be transferred to hepatocellular carcinoma cells (HCC), which resulted in decreased expression of stathmin-1 and insulin-like growth factor-1 receptor and inhibition of proliferation [57]. However, this transfer only occurred to a limited extent via exosomes, whereas the majority of miRNA transfer required intercellular contact and involved gap junctions. Recent results from investigations of exosome-mediated miRNA transfer between *Dicer*-proficient macrophages to *Dicer*-deficient endothelial cells equally only showed modest exosomal transfer [58]. Consequently, it is questionable that exosomal miRNA transfer alone could exert the observed biological effects in the aforementioned investigations. However, these results do neither exclude the potential for therapeutic effects from treatment with previously isolated and purified macrophage-derived exosomes nor the involvement of EVs in signal transduction of other cellular and tissue systems. For instance, the potentially deranged metabolism in cancerous cells might affect both packing of small RNA into EVs and the degree of their release. One recent exciting development in using EVs to modulate immune responses is based on helminth exosomes. Given the ability of helminths to exploit host immunity, the small RNAs and proteins in secreted helminth exosomes have been shown to suppresses the Th2 innate responses and eosinophilia against particular allergens, or suppress immune functions by inducing apoptosis and tolerogenic properties in DCs [59,60,61].

Overall the aforementioned reports all suggest that EVs play important roles in regulating the immune system *in vivo* and therefore have therapeutic potential against immune system-related diseases [62].

## 4. EVs as Drug-Delivery System Carriers

### 4.1. EVs as Carriers for Small Molecular Drugs

Different methods can be employed for loading small molecules into EVs. These include loading EVs after isolation from parental cells, loading parental cells with the drug, followed by isolation of released EVs, and transfecting parental cells with DNA encoding for the drug and subsequent isolation of the EVs. For instance, EVs loaded with doxorubicin have effectively been used to inhibit growth of breast and colon cancers [63,64]. These studies suggest that EVs can effectively deliver chemotherapeutics to treat malignant tumors.

Guo *et al.* showed that therapy with EVs purified from L1210 lymphocytic leukemia cell antigen/lipopolysaccharide-pulsed DCs and loaded with cyclophosphamide and poly(I:C) were able to induce DC maturation and most effectively suppress tumor growth and prolong survival time in tumor-bearing DBA2 mice [65]. These findings suggest that the combination of a tumor vaccine, a conventional anti-cancer agent, and a promoter of DC maturation might represent a generally useful strategy for anti-cancer therapy. MSCs have been proposed as delivery vehicles for anticancer agents because of their ability to migrate to tumor microenvironments. Pascucci *et al.* reported a new approach to drug delivery using EVs from MSCs. Following priming with paclitaxel (PTX), MSCs were able to strongly inhibit pancreatic tumors via their capacity to release the drug from PTX-loaded MSC EVs that were taken up by the cancer cells [66]. Tian and co-workers used a different approach to deliver doxorubicin (Dox) to tumors in BALB/c nude mice [63]. These authors loaded exosomes purified from immature dendritic cells (iDCs) with Dox via electroporation. Tumor targeting was enhanced by promoting expression of the exosomal membrane protein Lamp2b fused to αv integrin-specific iRGD peptide (CRGDKGPDC) in iDCs, whereas iDCs were used to reduce immunogenicity and toxicity. 

Interestingly, Federici *et al*. recently showed that the opposite to drug loading of EVs occurs in resistance to cytotoxic anti-cancer drugs, *i.e*., “drug unloading” of cisplatin [67]. Not only does extracellular acidosis impair cisplatin uptake by cancer cells *per se*, but also induces exosome-mediated elimination of cisplatin from cancer cells; both could be inhibited by addition of a proton-pump-inhibitor. 

### 4.2. EV-Encapsulated Curcumin

Curcumin, a natural polyphenol found in the rhizomes of *Curcuma longa* (turmeric), has been shown to exert various biological activities, including anti-inflammatory, anticancer, and antioxidant effects. However, due to its hydrophobicity, curcumin has poor aqueous solubility and a poor absorbability and overall bioavailability. To overcome these limitations, various delivery strategies have been used, including encapsulation in lecithin liposomes (small unilamellar vesicles) [68], micelles [69], cyclodextrins [70], and chitosan [71]. In addition, absorption enhancers, such as piperine have been used in conjunction with encapsulation to improve bioavailability [72].

Sun *et al.* on the other hand used EVs as a delivery vehicle for curcumin and showed that curcumin preferentially interacts with EVs in lipid membranes [73]. Additionally, curcumin delivered by EVs is more stable and more concentrated in blood, which indicates a significantly superior absorption and bioavailability. In a LPS-induced mouse model of septic shock, EV-encapsulated curcumin inhibited LPS-induced inflammation significantly more effectively than free curcumin [73]. This study provided evidence for the potential of EVs as drug-delivery carriers. Zhuang *et al.* improved the method of administration of EV-encapsulated curcumin: intranasal administration of EVs curcumin led to rapid delivery of curcumin to the brain and significantly inhibited brain tumor growth, LPS-induced brain inflammation, and experimental autoimmune encephalitis (EAE) in mouse models [74]. This strategy may provide a noninvasive and novel therapeutic approach for treating inflammatory brain diseases. In cancer therapy, Phase I and II clinical trials have yielded promising results on the use of curcumin as a component of pancreatic cancer therapeutic strategies [75]. In association with these data, EV-encapsulated curcumin also decreases the viability of pancreatic adenocarcinoma cell lines [76].

### 4.3. Nucleic Acid-Based Drugs and EVs

EVs carry multiple types of molecules including proteins and nucleic acids, and these cargoes are more stable than they would be if exposed to body fluids. In particular, mRNA and miRNA encapsulated in exosomes are called esRNA (exosome shuttle RNA). esRNA in body fluids such as serum, urine, and saliva is a biomarker for early diagnosis and surveillance of various disorders [77]. The fact that EVs can carry nucleic acids in stable form has attracted the attention of researchers who are developing nucleic acid-based drugs.

Alvarez-Erviti and co-workers developed a DDS for the central nervous system using modified EVs [20]. To reduce immunogenicity, they used autologous (self-derived) DCs for EV production. Targeting of nerve cells was achieved using a neuron-specific RVG peptide and loading of nucleic acid drugs was achieved by electroporation. siRNA delivered by intravenously injected RVG-targeted EVs can specifically inhibit target genes in the brain. The therapeutic potential of EV-mediated siRNA delivery was demonstrated by knockdown of BACE1; a therapeutic target in Alzheimer’s disease [20]. These results suggested that EVs can cross the blood-brain barrier and can be targeted by surface modification and loaded with nucleic acid drugs. We recently developed cancer-targeting EVs that inhibit progression of breast cancer in an *in vivo* mouse model [21]. Targeting was achieved by engineering the donor cells to express the transmembrane domain of platelet-derived growth factor receptor fused to the GE11 peptide; an artificial ligand of EGFR. The GE11-EVs delivered *let-7a* tumor suppressor microRNA and inhibited development of xenograft breast cancer cells in RAG2KO mice. Our results suggest that EVs can be used therapeutically to target cancer tissues with nucleic-acid drugs [21]. Additionally, MSC EVs-mediated transfer of *miR-133b* has been shown to promote neurite growth in neural cells [78]. Likewise, MSC-derived EVs loaded with *miR-146b* inhibit glioma growth [79]. Because MSCs are efficient producers of exosomes, they are well suited for mass production of exosomes, which are ideal for drug delivery [80]. Immune cells in particular are suitable target cells for EV-based therapeutic interventions. Bryniarski *et al.*, recently demonstrated effective T-cell inhibition via systemic transit of exosome-like nanovesicles delivering the inhibitory miRNA *miR-150* to target effector T-cells in an antigen-specific manner via surface coating with antibody light chains [81].

## 5. EVs in Clinical Trials

Because of the unique properties of EVs and overall encouraging preclinical results, it is not surprising that development of these vesicles as therapeutic agents is vigorously pressed on. Consequently, a number of clinical trials have been completed or are currently underway to determine their efficacy and safety as therapeutics or DDSs *in vivo* (Table 1). Phase I clinical trials have been conducted using tumor peptide/drug–loaded exosomes in patients with metastatic melanoma, advanced non-small cell lung cancer (NSCLC), colorectal cancer (CRC), and malignant glioma [82,83,84,85].

Advanced stage III/IV melanoma patients with tumors expressing the MAGE-3 antigen were enrolled and treated with MAGE-3 peptides (HLA-A1/B35) loaded dexosomes from autologous DCs in conjunction with MAGE-3 class II peptides (DP04) or tetanus toxin class II epitopes for helper effects [82]. Five out of the 15 enrolled patients experienced some measure of clinical benefit with respect to the primary tumor or lymph node lesions. Thirteen enrolled patients with pre-treated Stage IIIb and IV NSCLC expressing MAGE-A3 or A4 were treated with MAGE-A3, -A4, -A10, and DP04 peptides loaded dexosomes from autologous DCs [83]. Of the 13 enrolled patients, 9 completed the therapy and some patients benefited from long term disease stabilization and moderate immune effector activation, but overall no significant induction of MAGE peptide-specific T-cell responses could be monitored in patients. Consequently the authors engineered a new process for the isolation of second generation dexosomes with adjusted peptide formulation and improved immune stimulatory capacities [86]. A phase II follow-up with this improved vaccine is currently underway aimed at determining progression-free survival in 41 enrolled non-operable NSCLC patients after vaccination (Table 1). Forty patients with HLA-A*0201^+^CEA^+^ stage III or IV CRC were either treated with ascites-derived exosomes alone or in conjunction with granulocyte-macrophage colony-stimulating factor (GM-CSF) [84]. Thirty-seven patients completed the vaccinations and the results showed that only in patients receiving GM-CSF a beneficial anti-tumor cytotoxic T-cell response against colon cancer peptide CAP-1 could be observed. The fact that no significant effects were observed in patients who were treated with exosomes only shows that further preclinical research might be required to determine if sufficient immune inductive components are present in tumor-derived exosomes in general or if this was an exceptional case. A recent innovative pilot study used a vaccine based on autologous glioma tumor cells (isolated during surgical craniotomy), treated with an antisense oligodeoxynucleotide (AS-ODN) targeting insulin-like growth factor receptor-1 (IGF-1R), encapsulated in small diffusion chambers and re-implanted into the patient’s abdomen 24 h post-surgery for 24 h [85].

**Table 1:** Completed and running clinical trials of extracellular vesicle (EV)–based therapies.

Vesicle Type	Disease	Drug	EV Source	Admin.^1^	Patients	Therapeutic Results ^2^	Side-Effects ^2^	Status ^3^	Ref.
**Phase I clinical trials**									
Autologous dexosomes	Metastatic melanoma	Melanoma peptide antigens	DCs	i.d./s.c.	15	MART1–HLA-A2 T-cell response and tumor shrinkage (1); minor response (1); mixed response (1); stabilization (2)	No major toxicity; minor inflammation; grade 1 fever (5)	C	[82]
Autologous dexosomes	Non-small cell lung cancer	MAGE peptides	DCs	i.d./s.c.	9	MAGE-specific T cell responses (3); NK cell lysis (2)	No major toxicity; moderate pain (1), swelling at injection site (8); mild fever (1)	C	[83]
Autologous exosomes	Colorectal cancer	EVs, EVs + GM-CSF	Ascites fluid	s.c.	37 ^4^	EVs+GM-CSF: cytotoxic T cell resp. to CAP-1 (76.9%); stabilization (1); minor response (1)	No major toxicity; moderate pain, swelling, pruritus at injection site (37); fever (1), fatigue (3) and nausea (1)	C	[84]
Autologous exosomes	Malignant Glioma	EVs + AS-ODN	Glioma	Implanted biodiffusion chamber	12	N.D.	N.D.	C	[85]
Allogeneic exosomes/MVs	Type I diabetes mellitus	EVs	Umbilical cord-blood derived MSC	i.v.	20	N.D	N.D	E	[87]
Allogeneic exosomes	GVHD	EVs	MSC	i.v.	1	GVHD symptoms improved; stabilization for several months. Patient died of pneumonia 7 months post exosome application	No major side-effects	C	[35]
OMVs	Meningitis	Vaccine	B:4:P1.7-2,4; B:15:P1.7,16 strains	i.m.	91	MenBVac/MeNZB vaccines: high efficacy	No major toxicity; moderate pain, swelling, induration at the injection site; mild fever, malaise/headache	C	[88]
OMVs	Meningitis	Vaccine	FetA modified strain 44/76	i.m.	52	MenPF-1 vaccine:high additive efficacy	No major toxicity; moderate pain, swelling at injection site; mild fever, malaise, nausea/headache	C	[89]
Plexosomes ^5^	Colon cancer	Curcumin	Fruit	oral	35	N.D.	N.D.	R	[90]
Plexosomes ^5^	Mucositis	Curcumin	Fruit	oral	60	N.D.	N.D	R	[91]
**Phase II clinical trials**									
Autologous dexosomes	Non-small cell lung cancer	IFN-γ, MAGE peptides	DCs	i.d./s.c.	41	N.D	N.D	E	[86]
OMVs	Meningitis	rMenB vaccine, NadA/fHBP/NHBA	B:4:P1.7-2,4 strains	i.m.	147	rMenB+OMV: high additive efficacy	Moderate pain, swelling, induration at the injection site; mild fever; serious adverse events (18)	C	[92]

N.D.: No data; ^1^ Administration: i.d.: intradermal; s.c.: subcutaneous; i.m.: intramuscular; i.v.: intravenous; ^2^ Number of patients between brackets; ^3^ C: completed; E: evaluating; R: recruiting; ^4^ 13 patients also received GM-CSF; ^5^ Plant-derived exosomes; AS-ODN: antisense oligodeoxynucleotide targeting insulin-like growth factor receptor-1 (IGF-1R); fHBP: factor H binding protein; GM-CSF: granulocyte-macrophage colony-stimulating factor; GVHD: Graft-versus host disease; IFN-γ: interferon gamma; MSC: Mesenchymal stem cell; MVs: microvesicles; NadA: Neisserial adhesin A; NHBA: Neisserial heparin binding antigen; MenPF-1: FetA_on_PorA_on_.

The rationale is that AS-ODN loaded exosomes slowly diffuse out of the chamber and induce T-cell activation and immune memory. Although the study is listed as completed, no results have yet been published. However, the proposed methodology is currently being further developed in a pre-clinical setting [93,94] and the results of the clinical trial are forthcoming shortly (personal communication [95]). Treatment of a single patient suffering from GVHD as a consequence of allogeneic stem cell transplantation with peripheral blood stem cells for secondary acute myeloid leukemia with MSC-derived exosomes showed marked improvement of GVHD and stabilization of disease [35]. The authors isolated exosomes with anti-inflammatory profiles containing high quantities of IL-10, transforming growth factor beta and human leukocyte antigen-G. The only trail that does not target cancerous tissue is currently in phase I and aims to reduce inflammation and improve islets β-cells mass and glycemic control in patients with type I diabetes mellitus (T1DM) [87]. To this end, 20 enrolled patients are treated via intravenous infusion of cell free umbilical cord-blood derived MSC exosomes/MVs without additional modification with therapeutic agents. To conclude, the development of an ovarian cancer vaccine based on exosomes from the ascites fluid of ovarian cancer patients and the TLR3 agonist poly[I]:poly[C_12_U] (Ampligen^®^) is currently being prepared for phase I clinical testing [96].

Overall, the aforementioned phase I trials highlight the feasibility of large-scale EV production and show that administration of autologous EVs in particular is generally devoid of serious adverse side-effects, with one patient tolerating treatment as long as 21 months [82]. Reported side-effects included inflammatory responses at the site of injection and low-grade fever. None of the phase I trials reported serious toxicity or adverse immune responses. However, these trials were limited to vaccination attempts to induce or boost endogenous immunogenic responses to cancer antigens (Table 1) and at an early stage of evaluation, which makes drawing a definite conclusion regarding long-term safety premature.

In recent times, strategies were developed based on EVs from non-mammalian cells and were therefore not discussed above, but because this approach entered phase I clinical testing will briefly be included here, *i.e*., bacterial outer membrane vesicles (OMVs) and plant-based exosomes (plexosomes) as therapeutic carriers. OMVs have long been investigated in the context of anti-bacterial vaccines since OMVs are naturally released by Gram-negative bacteria and are part of their virulence [88,97]. A 2005 phase I trial showed that OMV-based vaccines from B:4:P1.7-2,4 and B:15:P1.7,16 NZ 98/254 meningococcal strains were 93%−97% effective against *Neisseria meningitidis* serogroup B and achieved significantly higher serum bactericidal titers than vaccines based on capsular polysaccharides [88,92]. A phase II follow-up trial was recently concluded in which 147 infants from the United Kingdom were immunized with either a recombinant vaccine (rMenB) containing Neisserial adhesin A (NadA), factor H binding protein (fHBP) and Neisserial heparin binding antigen (NHBA) alone or in combination with OMV from B:4:P1.7-2,4 (NZ 98/254) [92]. Both rMenB and rMenB+OMV were immunogenic against strains expressing homologous or related NadA and fHBP, but significantly less effective against heterologous fHBP variants. Overall, rMenB+OMV demonstrated greater immunogenicity and was also immunogenic against strains expressing homologous PorA. In addition, both vaccines stimulated immune memory (anamnestic) responses after the fourth dose and were overall well tolerated. Another approach to broaden the protective effect of OMV vaccines against *N. meningitidis* was tested by Marsay *et al*. based on ferric enterobactin receptor (FetA) and PorA expression (MenPF-1) in 52 adults [89]. The MenPF-1 vaccine was generated through genetic modification of *N. meningitides* strain 44/76 by replacing the promoter region of *fetA* with a 17 bp spacer region derived from the promoter region of *porA*, which resulted in constitutively expressed FetA and wild-type PorA. Their results show that FetA-specific reactions could be induced *per se* and that MenPF-1 (FetA_on_PorA_on_) showed broad and additive responses with titers ≥1:4 in 96%−100% of subjects after the final dose.

In the case of these OMV-based vaccines, immunogenicity is wanted. Gujrati and collaborators further developed OMVs as more general therapeutic vehicles by loading OMVs shed with a human epidermal growth factor receptor 2 (HER2)-specific affibody for active targeting from a mutant *E. coli* strain with siRNA targeting kinesin spindle protein (KSP) [98]. KSP silencing prevents the formation of bipolar mitotic spindles in rapidly dividing cells, which results in cell-cycle arrest and apoptosis [99,100]. In order to reduce endotoxicity and immunogenicity, the authors used a *msbB* mutant *E. Coli* strain that produces under-acylated (lacks the myristic acid moiety of lipid A) and shorter length LPS [101,102].

Results from the *N. meningitides* phase I clinical trials demonstrate that OMV-based vaccines induced no serious vaccine-related adverse events. Nonetheless, most patients displayed local reactions, including moderate pain, redness, swelling and induration at the injection site, whereas approximately 40%−50% of patients reported systemic reactions such as mild fever, malaise and headache [88]. Although the aforementioned modified OMV-based DDS displayed low immunogenicity and reduced toxicity as a consequence of the *msbB* mutant, safety concerns may still arise because of the bacterial origin of OMV-based therapeutics. Consequently, further clinical evaluation might be necessary when utilizing OMVs as vaccines, whereas OMVs that are used as DDSs require surface modification strategies and additional preclinical evaluation to meet particular safety criteria as therapeutics or DDSs.

Plexosomes have recently attracted attention because of their potentially low toxicity and immunogenicity‒plexosomes are consumed by humans on a daily basis‒and because they represent an unlimited source of DDS. Two phase I clinical trials have recently been initiated that predominantly evaluate if oral plexosome-based curcumin delivery to normal and cancerous colon tissue is more effective than with conventional DDSs [90] and if oral mucositis associated with chemoradiation treatment of head and neck cancer can be prevented [91]. Plant-derived EVs may solve isolation and purification restrictions, since plant-based EVs represent a potentially unlimited source of EVs for drug delivery.

## 6. EVs Caveats and Challenges

The application of EVs as therapeutics or DDSs has become an active area of research. However, many obstacles must still be overcome in order to successfully and safely use EVs as therapeutics in a routine clinical setting. The clinical trials that have been conducted at the phase I stage seem to indicate that no serious acute side effects occur. Nonetheless, those trials were limited to a particular biological action, *i.e.*, immune-modulation, and long-term experience is lacking. In the light of the many uncertainties regarding EVs, clinical trials with EVs, albeit attractive and highly desirable, should be performed with care. As deliberated in the introduction by Karla *et al*., [2] these uncertainties include a lack of unequivocal characterization and nomenclature based on cellular origin, size, physical properties, and unique markers, and a lack of suitable and standardized isolation methods. As a consequence of the aforementioned caveats, it is impossible to firmly determine EV classes, their biogenesis, physiological function, transport, how they reach their target cell, what their role in various disease processes is, whether EV networks should be targeted in therapeutics development and if therapeutics show additive, antagonistic, or synergistic effects, to name but a few. Furthermore, EVs from aberrant cells have been shown to carry the cell of origin’s divergent phenotype and therefore might carry tumorigenic potential [103,104,105,106]. Equally, pathogens have been shown to utilize EVs to spread infection and avoid the host’s immune system [107,108,109]. This clearly suggests that EVs may have an inherent disease-bearing potential, which justifies caution when isolating, manipulating or engineering EVs for therapeutic purposes. These are all safety concerns for which substantial precedence and experience exist with other types of biological therapeutics.

Given concerns about their immunogenicity, safety, and efficiency, EVs should be purified from self-derived and rapidly proliferating cells such as immune or induced pluripotent stem cells (iPS) for autologous treatment. Hu *et al.* successfully purified EVs from iPS cells-derived MSCs and showed that pretreatment with these EVs resulted in protection of limbs from ischemic injury in a mouse model [110]. Currently, because it would be very challenging to identify and understand all constituent elements of EVs, it is extremely difficult to realize clinical use of EVs. However, EVs are easier to apply clinically than iPS cells because they are not intact cellular material and consequently have a lower potential for malignant alterations. Furthermore, transplantation of self-derived MSCs and DCs has extensively been investigated in many clinical trials. Therefore, the safety issue regarding severe adverse immunogenic responses as a result of the administration of EVs purified from patient-derived MSCs and DCs has essentially already been resolved.

To manufacture EVs as medicines, it will be necessary to establish methods for stable production and bulk preparation. Jang *et al.* developed bioinspired exosome-mimetic nanovesicles from monocytes or macrophages by serial extrusion through filters with diminishing pore sizes (10, 5 and 1 μm) [64]. Such a method represents an effective way to ensure sufficiently high quantities of exosomes for clinical use. If self-derived EVs are to be used for therapy, it will be necessary to develop a system for personalized medicine. The most common method for EV purification involves ultracentrifugation, but this method is not well suited for bulk preparation. Consequently, novel isolation, separation and refinement technologies capable of sufficiently high yields are urgently required.

The function and the treatment effect of the purified EVs are mainly summarized in this review. On the one hand, the physiological function of EVs in several diseases may be therapeutic targets. Because some pathways regulate the secretion machinery of EVs such as ceramide and the ESCRT machinery [111,112,113], cell and mouse models completely lacking EVs have not been created. Therefore, the physiological significance *in vivo* and involvement of EVs in various diseases remains unclear. Nonetheless, recent research is increasingly implicating EVs in particular disorders. For instance, the accumulation of pathological tau protein, a major hallmark of Alzheimer’s disease, was shown to be promoted via EVs derived from microglia [114]. In addition, inhibition of the ESCRT machinery decreased infectious prion release [115]. Exosomes from HCV-infected cells were shown to be capable of transmitting infection to naive human hepatoma cells and able to establish a productive infection by exosome-mediated transmission of HCV RNA [116]. These results suggest that the inhibition of EV secretion pathways and/or the depletion of EVs from blood from the blood of patients through apheresis may represent a novel strategy to modulate or interrupt disease progression.

## 7. Conclusions

In recent years, the number of EV-related studies has increased rapidly, and specialized journals and conferences have been established to disseminate these findings. The knowledge about EVs obtained from research of multiple life science disciplines shows great potential for various applications, particularly in regenerative medicine and to treat diseases refractory to conventional approaches. EVs may be considered to be the smaller “alter egos” of secretory cells and, therefore, may function equivalent to implantation of secretory cells; at least partially. However, compared to living cell transplantation, administration of EVs will decrease the burden on both physicians and patients with regard to production, quality management and therapy cost. Thus, one important characteristic of EVs in the context of therapeutic applications is that they might replace cell transplantation in the long run. On the other hand, the DDS field has focused on other characteristics of EVs. To date, the development of DDS has been hampered by biological toxicity and antigenicity of carriers. The ability of EVs to deliver molecules to distant cells via body fluids without significant toxicity and antigenicity demonstrates their utility as DDS. Nonetheless, further studies are needed to establish EVs as the next-generation DDSs or novel biological drugs. Additional information about various aspects of extracellular vesicles is provided in the other reviews in this focus edition [2,117,118,119,120].
